# Effects of virtual interventions based on the theory of planned behavior to improve obesity-preventive lifestyle among girls, during COVID-19 pandemic

**DOI:** 10.1186/s12889-023-17259-2

**Published:** 2023-11-24

**Authors:** Rafat Moghimi, Mostafa Nasirzadeh, Hassan Ahmadinia, Azizollah Pourmahmoudi, Mahdi Abdol Karimi

**Affiliations:** 1 Department of Health Education and Health Promotion, Faculty of Health, Rafsanjan University of Medical Sciences, Rafsanjan, Iran; 2 Department of Health Education and Health Promotion, School of Health, Occupational Health and Safety Research Center, NICICO, World Safety Organization, Rafsanjan University of Medical Sciences, Rafsanjan, Iran; 3 Department of Epidemiology and Biostatistics, Faculty of Medicine, Rafsanjan University of Medical Sciences, Rafsanjan, Iran; 4 Department of Nutrition and Food Sciences, Faculty of Health and Nutrition, Yasuj University of Medical Sciences, Yasuj, Iran; 5 Department of Health Education and Health Promotion, Faculty of Health, Geriatric Care Research Center, Rafsanjan University of Medical Sciences, Rafsanjan, Iran

**Keywords:** Obesity, Nutrition, Physical activity, Planed behavior theory, Covid-19

## Abstract

**Background:**

Adolescence is a critical period for the spread of obesity and overweight. This research was conducted with the aim of determining the effect of an educational intervention based on the theory of planned behavior on promoting obesity-related behaviors in overweight female students in Gachsaran.

**Methods:**

this quasi-experimental study was conducted on 90 female students of the first secondary school in the form of two intervention and control groups. Information related to nutritional status and the structures of the theory of planned behavior were collected using a researcher-made questionnaire whose validity and reliability have been confirmed. The educational intervention was carried out during five virtual training sessions. The data obtained three months after the intervention were analyzed using SPSS statistical software, version 20, using independent t-tests, paired t-tests, and equivalent non-parametric tests.

**Results:**

The present study showed that the scores of the constructs of awareness, perceived behavior control, subjective norms, intention, and nutritional behaviors were significantly improved after the intervention (p < 0.001). The results of the Mann-Whitney test showed that the two intervention and control groups did not have a significant difference in terms of the average overall physical activity score after the intervention (p = 0.078).

**Conclusion:**

The results of the present study showed that training based on the theory of planned behavior in the conditions of COVID-19 disease and in a virtual form had an effect on nutritional behavior but could not increase physical activity behavior in adolescents with weight loss.

**Supplementary Information:**

The online version contains supplementary material available at 10.1186/s12889-023-17259-2.

## Introduction

One of the most basic public health problems in developed and developing countries is the increasing prevalence of obesity and overweight in children and adolescents [1]. The significant increase in the prevalence of childhood obesity during the last few decades has changed existing attitudes about childhood obesity, and this issue has been considered one of the 10 most important health problems globally [2]. Childhood obesity is now recognized as one of the most serious challenges of the 21st century [3]. According to the definition by the Centers for Disease Control and Prevention, an overweight and obese child is defined as a child whose body mass index is at or above the 85th percentile for their age [4]. It is predicted that the phenomenon of childhood obesity will increase to 60% in the next decade, affecting 250 million children by 2030 [5]. In Iran, the highest prevalence of obesity is observed in adolescents [6]. Obesity and overweight during adolescence can increase the risk of chronic diseases in adulthood [7]. The impact of obesity is evident in three aspects: the persistence of obesity into adulthood, increased risk for diseases such as type 2 diabetes, cardiovascular disease, chronic kidney disease, and cancer, as well as increased mortality and premature death. Overweight and obesity, as well as related diseases, can largely be prevented [3]. The most important factors considered as determinants of obesity in children are an unhealthy diet and lack of physical activity [8].

Based on the World Health Organization’s recommendation, children and adolescents aged 5 to 17 should engage in at least 60 min of moderate-to-vigorous intensity physical activity daily [9]. However, the majority of adolescents do not meet the current physical activity guidelines [10]. Several studies have shown that most overweight adolescents do not adhere to the recommended dietary guidelines [11].

In terms of inappropriate nutritional patterns, the main cause of obesity is prolonged energy imbalance resulting from excessive energy intake compared to energy expenditure. Studies indicate that the consumption of key foods among adolescents, such as fruits, vegetables, and milk/dairy products, falls below the recommended levels, while the consumption of meat/meat products exceeds the recommended levels [12].

The COVID-19 pandemic has led to a rise in obesity cases among children and adolescents. During the pandemic, school closures and disruptions in their lives affected more than 80% of children, leading to increased social isolation and a higher risk of deteriorating health and obesity in adolescents [13]. Studies indicate that eating patterns deteriorated during the pandemic, with many children and adolescents consuming unhealthy foods like snacks and overeating [14].

Moreover, the closure of schools during the COVID-19 pandemic resulted in reduced physical activity. Online classes increased children’s sedentary time from five hours to 8 to 10 h [15]. To prevent the increased prevalence of obesity during and after the epidemic, it is crucial to promote a healthy lifestyle. Effective education serves as the initial step in changing unhealthy behaviors. Previous studies have shown the usefulness of health education theories as the foundation for programs targeting overweight school students [16]. One such theory is the theory of planned behavior, which emphasizes behavioral intention as the strongest determinant of behavior. This intention is influenced by attitude towards behavior, subjective norms, and perceived behavioral control [17]. Interventions based on the theory of planned behavior focus on three parts: attitude (positive or negative assessment) toward the behavior, subjective norms (perception of significant others’ thoughts), and perceived behavioral control (perception of the amount of control on behavior implementation.Several studies have reported the effectiveness of the theory of planned behavior constructs in predicting healthy eating behaviors and physical activity in various populations [18, 19].Women are at higher risk for developing obesity-related comorbidities as compared to men and have a twofold higher mortality risk than overweight men, and intervention to prevent overweight in them is very important [20].

Considering the prevalence of overweight and obesity among adolescents, sedentary lifestyles, and inappropriate nutrition during the COVID-19 pandemic, performing education interventions based on theory is essential to improve these behaviors.

During the disease epidemic, due to the absence of students in schools and the risks associated with face-to-face teaching, theory-based interventions were designed and implemented in the form of virtual training. Therefore, the aim of this research is to evaluate whether an educational intervention based on the theory of planned behavior in virtual sessions improves physical activity behavior and proper nutrition in adolescent girls suffering from overweight and obesity.

## Methods

The present study was a quasi-experimental research conducted to investigate the effect of an educational intervention based on the theory of planned behavior on behaviors related to obesity and overweight in 90 first-grade female high school students (students from 7th class to 9th class) in Gachsaran city in 2019–2020. According to the sample size formula, considering a similar study [21] with a 95% confidence interval, statistical power of 90%, and a standard deviation of attitude score of 3.7, 40 participants were estimated for each group. Considering the possibility of attrition, 45 participants were included in the study. The students were selected using a multi-stage random sampling method. Firstly, four schools were randomly selected from a total of 24 girls’ high schools, and out of these four schools, two were randomly assigned as the intervention group and two as the control group. The sample consisted of 45 participants in the intervention group and 45 participants in the control group, randomly selected from each grade (first, second, and third) based on the inclusion criteria. Overweight students in each group were selected by lottery method. With the cooperation of school health care workers, a list of overweight and obese students was prepared, and an equal number of students from schools and grades were randomly selected to enter the study. All parents received written information about the questionnaire and procedure. Informed written consent will be obtained from all students and their parents. For parents who were illiterate, since risks associated with research were low, after explaining the objectives of the intervention in a language understandable to them, oral informed consent was obtained, this protocol was approved by the ethics committee of Rafsanjan University of Medical Sciences.

The inclusion criteria were being in the 85th percentile and above based on the growth chart of the World Health Organization, parental satisfaction, absence of physical disabilities, heart disease, respiratory disease, mental illness, and certain systemic diseases such as hypothyroidism and Cushing’s. The evaluation of these diseases was conducted by examining the students’ health records and obtaining information through questioning.

The exclusion criteria included student migration, new disease diagnosis, absence in more than two sessions, and non-cooperation in completing the questionnaire. The data collection tool is a questionnaire based on the constructs of the theory of planned behavior regarding two behaviors related to obesity: nutritional status and physical activity. The validity and reliability of this questionnaire have already been examined and confirmed [21].

The reliability of the questionnaire was confirmed by determining the internal consistency using Cronbach’s alpha coefficient (0.78) through a preliminary study involving 35 participants.

This questionnaire consists of seven parts. The first part includes demographic questions such as age, height, weight, body mass index, educational level, parents’ education level, parents’ occupation, and family income. Classification of social variables was done based on common education levels in the study population and income level was qualitatively classified.

The second part includes questions related to measuring knowledge, which consists of 10 questions. The minimum and maximum score for knowledge can range from 0 to 20.The next part focuses on the constructs of the theory of planned behavior, including 20 questions for attitude, 12 questions for subjective norms, 10 questions for perceived behavioral control, 7 questions for behavioral intention, and 10 questions for behavior. These questions were measured using a 5-point Likert scale.

To measure physical activity behavior, the short form of the International Physical Activity Questionnaire (IPAQ) was utilized. This questionnaire has been used in various studies, and its validity and reliability have been confirmed [22]. To assess the reliability of the International Physical Activity Questionnaire in this group, the test-retest method was employed. The questionnaire was distributed among 25 students, and after the initial test and a follow-up test two weeks later, the correlation coefficient was found to be 0.82, indicating good reliability of the questionnaire.

For the initial evaluation of educational needs based on the theory of planned behavior, the electronic pre-test questionnaire was completed and analyzed. Educational goals were established, encompassing five subjects: raising awareness, creating and improving a positive attitude towards obesity prevention behaviors, training managers, teachers, and parents of students, training focused on improving perceived behavioral control and behavioral intention for greater impact on behavior.

The educational intervention was designed and implemented in a virtual format, consisting of four sessions for students (each lasting 50–60 min) and one session for parents, managers, and teachers (also lasting 50–60 min). Various relevant educational methods were employed, including lectures, podcasts, group discussions, introduction of sports and nutrition-related blogs and websites, presentation of video clips and educational videos, sharing related photos, brainstorming, role-playing, distribution of educational pamphlets, SMS communication, and question-and-answer sessions (Table 1).

**Table 1:** Educational content of intervention group meetings

Meeting	Objectives of the meeting	Summary of activities
**First**	Promoting the awareness of student for obesity prevention behavior.	Explaining the effects of weight gain on health. Getting to know the principles of proper nutrition and types of physical activity
**Second**	Creating and improving a positive attitude towards obesity prevention behaviors	Promoting positive attitudes toward weight-loss behaviors by discussing misconceptions about obesity and physical activity and nutrition in adolescence.
**Third**	Promoting social norms and for control and prevention of obesity	Modifying the beliefs of people who influence teenagers, in the field of obesity prevention behaviors, such as family members, school health coaches and school administrators.
**Fourth**	Promoting the perceived behavioral control of student in the field of prevention of obesity	Promoting the feeling of empowerment and control over performing obesity prevention behaviors (physical activities, healthy eating) was discussed and solutions were presented to promote the feeling of empowerment.
**Fifth**	Greater increase in the intention to perform obesity prevention behaviors	Inviting the health officer of education schools, school managers and instructors in the group of students and sending educational content to students through virtual groups.

After 3 months, we conducted the post-test by distributing the same questionnaires to both the intervention and control groups, following the same procedure as the pre-test. Questionnaires were presented on the Porsline, an online survey platform in Iran. After explaining the objectives of the research and emphasizing the confidentiality of the information and obtaining informed consent from the parents, we posted the online survey link on Telegram and WhatsApp, two of Iran’s most widely used social media platforms and Questionnaires were completed virtually. In order to prevent bias, the schools included in the study were asked to avoid unintended interventions or co-interventions.

The data were analyzed using SPSS-22 software, employing independent and paired t-tests, chi-square tests, and Analysis of Covariance (ANCOVA). The normality of the data was assessed using the Kolmogorov-Smirnov test. Nonparametric statistics were used if the data did not follow a normal distribution.

## Results

The age range of students in the control and intervention groups was 12–16 years. Mean age of the participants were (13.96 ± 1.03).

The chi-square test results conducted before the educational intervention indicated no statistically significant differences between the intervention and control groups in terms of all demographic variables. The analysis of variance conducted before the educational intervention revealed no statistically significant differences in the score of obesity prevention behaviors based on demographic characteristics (P < 0.05) (Table 2).

**Table 2:** Comparison of obesity prevention behaviors of students according to demographic characteristics

Variable		Frequency (%)	Mean ± SD	*p* value
**Father education**	Illiterate	1(1.1)	22	0.86
	Primary school	2(2.2)	22.6 ± 6.42	
	Middle school	5(5.5)	20.7 ± 6/20	
	Diploma	37(41.1)	23.6 ± 6.27	
	Academic	45(50)	24.5 ± 8.75	
**Father job**	Employee	44(48)	22.6 ± 8.53	0.40
	Manual worker	10(11.11)	23.4 ± 6.43	
	Retired	8(8.88)	19.8 ± 7.43	
	Self-employment	26(28.88)	23 ± 9.74	
	Unemployed	2(2.22)	18.7 ± 7.76	
**Mother education**	Iliterate	2(2.22)	24 ± 4.4	0.67
	Primary school	7(7.77)	23.7 ± 8.95	
	Middle school	11(12.22)	25.4 ± 5.5	
	Diploma	43(47.77)	22.1 ± 9.25	
	Academic	27(30)	21.3 ± 9.23	
**Mother job**	Housewife	21(23.3)	22.4 ± 8.69	0.92
	Employee	69(76/66)	22.6 ± 8.93	
**Income level**	Low income	36(40)	23.25 ± 9.58	0.30
	Moderate income	29(32.2)	20.41 ± 7.65	
	Good income	25(27.7)	23.20 ± 9	

Based on the results of the independent t-test, there was no significant difference in body mass index (BMI) between the two groups before and after the intervention (p > 0.005). However, the paired t-test results showed a significant decrease in BMI in the intervention group compared to the control group (p = 0.004).Before the educational intervention, there were no significant differences in the scores of the theory of planned behavior constructs between the two groups. However, after 3 months, the mean and standard deviation of the scores for awareness, perceived behavior control, subjective norms, intention, and nutritional behaviors significantly improved in the intervention group (p < 0.001). Although the attitude score had increased, this difference was not statistically significant (p = 0.075) (Table 3).

**Table 3:** Comparison of the average scores of the constructs of the theory of planned behavior before and after the intervention in two groups

Variable	group	Before the intervention (n = 86) Mean ± SD	3 months after the intervention (n = 86) Mean ± SD	*p*-value**
**Knowledge**	Intervention (n = 45)	7.80 ± 2.389	18 ± 2.088	P < 0.0001
	Control (n = 45)	7 ± 2.215	7.22 ± 2.214	0.417
	*p*-value*	0.103	P < 0.0001	
**Attitude**	Intervention (n = 45)	77.53 ± 10.154	79.51 ± 5.81	0.272
	Control (n = 45)	80.60 ± 7.98	80 ± 8.54	0.508
	*p*-value*	0.115	0.752	
**Subjective norms**	Intervention (n = 45)	44.48 ± 7.85	50.73 ± 5.36	P < 0.0001
	Control (n = 45)	46.26 ± 8.13	45.84 ± 8.33	0.602
	*p*-value*	0.295	0.001	
**Perceived behavioral control**	Intervention (n = 45)	30.71 ± 9.28	36.93 ± 7.09	P < 0.0001
	Control (n = 45)	33.71 ± 8.73	32.57 ± 6.60	0.203
	*p*-value*	0.118	0.003	
**Intention**	Intervention (n = 45)	27.60 ± 6.443	31.02 ± 3.194	0.002
	Control (n = 45)	27.70 ± 5.398	27.95 ± 6.136	0.747
	*p*-value*	0.934	0.001	
**Behavior**	Intervention (n = 45)	23.04 ± 8.604	33.08 ± 7.612	0.044
	Control (n = 45)	21.88 ± 8.858	22.66 ± 8.087	0.347
	*p*-value*	0.532	0.000	
**Body Mass Index**	Intervention (n = 45)	26.16 ± 3.51	25.44 ± 4.186	0.004
	Control (n = 45)	25.95 ± 1.618	25.99 ± 1.568	0.296
	*p*-value*	0.715	0.418	

* Independent T-test

** Paired-t test

The results of the Analysis of Covariance (ANCOVA) demonstrated a significant difference in the constructs of awareness, perceived behavior control, subjective norms, intention, and nutritional behaviors after the educational intervention (p < 0.005). However, the difference in attitude score was not statistically significant (p = 0.795) (Table 4).

**Table 4:** Results of analysis of covariance to investigate changes in the level of knowledge and constructs of the theory of planned behavior for obesity prevention behavior in students

Variable		DF	mean square	F	*p* value
**Knowledge**	Group	1	2404.27	598.48	P < 0.0001
	Pre-test	1	58.27	14.50	P < 0.0001
	Error	87	349.50		
**Attitudes**	Group	1	3.21	0.068	0.795
	Pre-test	1	593.65	12.58	0.001
	Error	87	4105.558		
**Subjective norms**	Group	1	711.61	18.97	P < 0.0001
	Pre-test	1	1056.74	28.18	P < 0.0001
	Error	87	3261.96		
**Perceived behavioral control**	Group	1	556.61	111.09	P < 0.0001
	Pre-test	1	376.33	12.89	0.004
	Error	1	3755.44		
**Intention**	Group	87	556.61	12.84	0.001
	Pre-test	1	376.33	P < 0.0001	0.994
	Error	1	3755.44		
**Behavior**	Group	87	2399.61	38.64	P < 0.0001
	Pre-test	1	26.05	0.42	0.519
	Error	1	62.08		

Df: Degrees of freedom

The results of the chi-square test indicated that there was no statistically significant difference in the level of physical activity between the intervention and control groups before the educational intervention (p = 0.304). Similarly, after 3 months, there was no significant difference between the two groups (p = 0.393).

Due to the non-normal distribution of physical activity data, the Mann-Whitney test was used to compare the mean scores of intense, moderate, and weak physical activity between the intervention and control groups before and after the intervention. The results showed no significant difference in physical activity levels between the two groups. The Wilcoxon test also revealed no significant difference in physical activity levels within each group before and after the intervention (Table 5).

**Table 5:** Comparison of mean and standard deviation of the physical activity score of research samples in the intervention and control groups

Variable	**group**	Before the intervention		after the intervention		*p* value Wilcoxon
		Mean ± SD	(Median IQR)	Mean ± SD	(Median IQR)	
**severe physical activity**	Intervention	1408.39 ± 1115.56	640 (16.80)	1535.09 ± 1255.11	720 (15.60)	0.940
	Control	1283.38 ± 1175.47	960 (1300)	11660.74 ± 1447.77	960 (1600)	0.533
***p*** ** value Mann-Whitney**		0.533		0.174		
**Moderate physical activity**	Intervention	428.97 ± 336.44	180)480 (	352.00 ± 499.58	200(360)	0.859
	Control	612.14 ± 532.11	360 (698)	797.14 ± 698.66	480 (840)	0.127
***p*** ** value Mann-Whitney**		0.113		0. 141		
**walk physical activity**	Intervention	664.82 ± 596.93	445 (825)	575.99 ± 481.06	297 (554)	0.327
	Control	604.97 ± 705.83	721 (990)	539.23 ± 628.27	495 (742)	0.746
***p*** ** value Mann-Whitney**		0.217		0.056		
**Overall physical activity**	Intervention	2160.85 ± 2048.93	1448 (2467)	2020.20 ± 1911.37	1313 (2020)	0.74
	Control	1828.80 ± 2413.41	2034 (1959)	1921.69 ± 2416.53	2076 (2196)	0.95
***p*** ** value Mann-Whitney**		0.092		0.078		

## Discussion

This research was a quasi-experimental intervention study conducted to investigate the effect of an educational intervention based on the theory of planned behavior on promoting obesity-related behaviors in first-grade overweight female high school students in Gachsaran City.

In this study, there was no significant relationship between income level and obesity prevention behaviors, which contradicted the findings of Kim et al.‘s study [23].

Since most participants had low or moderate income levels, the influence of income level on obesity prevention behaviors may not have been fully explored. Following the educational intervention, a significant decrease in body mass index (BMI) was observed, which aligns with the findings of Sanaeinasab et al.‘s study demonstrating a significant reduction in BMI after an educational intervention. It appears that interventions based on the theory of planned behavior are moderately effective in promoting weight loss [24]. In recent study, the nutritional behaviors of teenagers in the intervention group were modified, resulting in reduced consumption of high-calorie snacks and increased consumption of fruits and vegetables. Studies in this area have shown that modifying dietary patterns in children and adolescents, along with weight control, can be achieved through targeted health promotion programs [25]. In our study, the intervention had a significant effect on the scores of all constructs of the theory of planned behavior, except for attitude. The findings revealed a significant increase in the awareness score among the intervention group three months after the intervention, which aligns with the results of Sheikh Ahmadi et al.‘s study [26]. However, there was no statistically significant difference in the average attitude score before and after the educational intervention, which contradicts Ahmadi et al.‘s study on promoting healthy lifestyle behaviors among students [27]. This finding is consistent with Peyman et al.‘s study on the consumption of low-value snacks among students, where no significant change was observed in the attitude score [28].

The variation in results across studies may be attributed to differences in educational approaches and initial attitudes within the target group. One possible reason for the non-significance of the attitude score in our study could be the COVID-19 pandemic, which led to online and virtual interventions. Attitude change is often more effectively accomplished through focused and face-to-face group discussions. Therefore, it is necessary to design and implement more effective educational programs to change the attitudes of teenagers.

The present study demonstrated a positive impact on subjective norms related to obesity prevention behaviors, which is consistent with the results of Market et al.‘s [29] and Khairi et al.‘s studies [30] but contradicts Barati et al.‘s study [31]. It should be noted that the influence of subjective norms can vary across different cultures. For instance, in some cultures, subjective norms are influenced by friends, while in others, they are mainly influenced by family [32].

Furthermore, the perceived behavioral control score of the students regarding physical activity and healthy eating behaviors improved after the educational intervention, which is in line with Shing et al.‘s study [33]. Generally, people are motivated to engage in health behaviors and are prepared to overcome the challenges associated with these behaviors when they have a clear understanding of the difficulty or ease of performing them and when they feel a sense of control over those behaviors [34]. The intention to engage in obesity-preventing behaviors also improved in the intervention group, which is consistent with Darabi et al.‘s study [35].

Nutritional behaviors improved among students in the intervention group, indicating the positive effects of the intervention on their dietary habits. This finding is consistent with Hockman et al.‘s study on diet-based nutritional interventions among teenagers [36]. However, there are inconsistent results in studies, such as Kote et al.‘s study, where theory-based education did not change behavior scores [37]. There is limited research on theory-based virtual training for lifestyle modification, but Barkhordari’s study showed that virtual lifestyle training is effective in promoting self-care behaviors [38]. However, it is necessary to compare the effectiveness of virtual and face-to-face interventions.

The results of the present study revealed no statistically significant difference in physical activity scores between the two groups before and three months after the educational intervention. This finding is consistent with Ahmadi Tabatabai et al.‘s study [28] but inconsistent with Mirzaei et al.‘s study, which showed an improvement in physical activity scores [39]. It should be noted that various studies worldwide emphasize that motivation and attitude are important predictors of participation in physical activities [40]. In our study, there was no significant change in attitude within the intervention group, and we did not investigate the motivational factors in our interventions. Physical activity is directly related to factors such as access to suitable exercise facilities, equipment, and social and family support [41]. Considering that during the pandemic, these access points have been limited, it can affect the study results.

This study is one of the few that specifically investigated the effect of theory-based interventions on obesity prevention behaviors in a high-risk target group during the COVID-19 pandemic. However, it had some limitations. One limitation was the measurement of behavior through self-report, which can affect the quality of the results. While self-report questionnaires are commonly used in health studies to assess nutritional status, recent research suggests that these tools may not always provide accurate results, especially regarding nutrition [42]. Another limitation is that the study was conducted in a limited population and only in girls, making it difficult to generalize the results. It is recommended to conduct future studies with larger and more diverse populations to compare the effects of interventions based on this model in both virtual and face-to-face settings.

## Conclusion

The findings of the present study demonstrate that educational interventions based on the structures of the theory of planned behavior, even in virtual settings during critical situations like the COVID-19 pandemic, can lead to significant improvements in awareness, subjective norms, perceived behavioral control, behavioral intention, and nutritional behaviors among overweight girls. However, there was no significant improvement in attitude and physical activity levels. Given the complex nature of physical activity behavior, it is necessary to design health promotion interventions that create the required environmental and social conditions to increase physical activity levels among adolescents with weight gain.

### Electronic supplementary material

Below is the link to the electronic supplementary material.

Supplementary Material 1

Supplementary Material 2

## Data Availability

The data underlying this article will be shared on reasonable request to the. corresponding author.
