# Supplementary material for: Effects of virtual interventions based on the theory of planned behavior to improve obesity-preventive lifestyle among girls, during COVID-19 pandemic
Source: BMC Public Health. 2023 Nov 24;23:2332. doi: 10.1186/s12889-023-17259-2 (PMC10675936; doi:10.1186/s12889-023-17259-2)
Supplement: Supplementary file 1 — Supplementary Material 1 [file 12889_2023_17259_MOESM1_ESM.docx]

**Weight loss questionnaire for students**

|  | **Questionnaire code:** |
| --- | --- |
| **HELLO dear student**  This questionnaire has been designed in order to carry out a research thesis in the field of investigating the effect of education on weight loss of overweight and obese teenagers. The information of this questionnaire is kept confidential and will only be used for research purposes. The success of this project requires accurate and honest answers from you dear teenagers. Thank you very much for helping us in this research. | |
| First part: Demographic profile: | |
| 1.Age……year 2.mobile number….. 3.weight…..kg 4.height….cm 5.Body mass index (BMI)…….. | |
| 6.What level of education are you studying in?  Frist secondary level (the seventh)🞎 Frist secondary level )Eight) 🞎 Frist secondary level (ninth) 🞎 | |
| 7. Your mother's education level ?  illiterate🞎 elementary 🞎 cycle🞎 diploma 🞎associate degree 🞎 Bachelors degree and higher🞎 | |
| 8. Your father's level of education ?  illiterate🞎 elementary 🞎 cycle🞎 diploma 🞎associate degree 🞎 Bachelors degree and higher🞎 | |
| 9.mothers job? House keeper🞎 employee 🞎  10. fathers job ? manual worker🞎 employee 🞎 retired 🞎 free job 🞎 unemployed 🞎 etc🞎 | |
| 11. What is the household income per month?  Less than 30 million Iranian rials 🞎 Between 30 and 50 million Iranian rials 🞎Between 50 million and 80 million rials 🞎 | |
| 12. Do you think you are overweight or obese? yes🞎 No🞎 | |
| 13. Do you have access to a computer or mobile phone at home? yes🞎 No🞎 | |
| 14. How many hours do you watch TV a day? less than an hour🞎 1 hours to 3 🞎 more than 3 hour🞎 I don’t watch | |

**BMI will be calculated by the researcher**

**Part 2 :Awareness Questions**

**Please mark your opinion on the following items**

**لطفا نظر خود را در زمینه موارد زیر علامت بزنید.**

| 1.Due to their high mental and physical activity, teenagers should use sugary foods in between their meals so that they do not get weak.  Right 🞎 wrong 🞎 I don’t know 🞎 |
| --- |
| 2.Foods such as bread and rice form the main part of teenagers' meals.  Right 🞎 wrong 🞎 I don’t know 🞎 |
| 3.A low-fat diet is enough to lose weight.  Right 🞎 wrong 🞎 I don’t know 🞎 |
| 4.Eating breakfast regularly can reduce weight in teenagers.  Right 🞎 wrong 🞎 I don’t know 🞎 |
| 5.Excess weight at the age of 30 years old causes diseases such as diabetes and blood pressure.  Right 🞎 wrong 🞎 I don’t know 🞎 |
| 6.The use of prepared foods in teenagers is the most important reason for overweight.  Right 🞎 wrong 🞎 I don’t know 🞎 |
| 7.Consuming one fruit or one glass of vegetables per day will help you lose wight.  Right 🞎 wrong 🞎 I don’t know 🞎 |
| 8.Walking for an hour a day is enough for teenagers to lose weight .  Right 🞎 wrong 🞎 I don’t know 🞎 |
| 9.The amount of calories from starchy foods is higher than other food groups.  Right 🞎 wrong 🞎 I don’t know 🞎 |
| 10.Vigorous physical activity is an activity that causes the heart rate and breathing to be faster than normal.  Right 🞎 wrong 🞎 I don’t know 🞎 |

| Please read the following questions carefully and comment on each of the following sentences | |
| --- | --- |
| **quite agree🞎 agree 🞎 No idea 🞎 disagree 🞎 quite disagree 🞎** | **1.A weight loss reduces my risk of cardiovascular disease.** |
| **It is very important🞎 important 🞎 No idea 🞎**  **its not important 🞎 it is not realy important🞎** | **2.In my opinion, prevention of cardiovascular diseases…** |
| **quite agree🞎 agree 🞎 No idea 🞎 disagree 🞎 quite disagree 🞎** | **3. In my opinion, eating low-calorie or healthy foods helps me lose weight** |
| **It is very important🞎 important 🞎 No idea 🞎**  **its not important 🞎 it is not realy important🞎** | **4. In my opinion, having a normal weight** |
| **quite agree🞎 agree 🞎 No idea 🞎 disagree 🞎 quite disagree 🞎** | **5.In my opinion, reducing the time of using TV, computer, tablet and mobile phone has a great effect on my weight loss** |
| **It is very important🞎 important 🞎 No idea 🞎**  **its not important 🞎 it is not realy important🞎** | **6.Reducing the time of using TV, tablet and mobile phone to reach the ideal natural weight** |
| **quite agree🞎 agree 🞎 No idea 🞎 disagree 🞎 quite disagree 🞎** | **7.Doing regular physical activities such as cycling, walking, swimming makes me lose weight** |
| **It is very important🞎 important 🞎 No idea 🞎**  **its not important 🞎 it is not realy important🞎** | **8.Doing regular physical activities to lose weight** |
| **quite agree🞎 agree 🞎 No idea 🞎 disagree 🞎 quite disagree 🞎** | **9.I lose weight, it becomes easier and more enjoyable for me to do physical activities, play, run, walk, etc.** |
| **It is very important🞎 important 🞎 No idea 🞎**  **its not important 🞎 it is not realy important🞎** | **10. In my opinion, doing physical activities without feeling tired** |
| **quite agree🞎 agree 🞎 No idea 🞎 disagree 🞎 quite disagree 🞎** | **11.I think losing weight makes me more attractive or Stylish to others** |
| **It is very important🞎 important 🞎 No idea 🞎**  **its not important 🞎 it is not realy important🞎** | **12.I get teased by others as I reach my normal weight and lose less weight** |
| **quite agree🞎 agree 🞎 No idea 🞎 disagree 🞎 quite disagree 🞎** | **13.Not being ridiculed by others for being overweight and obese** |
| **It is very important🞎 important 🞎 No idea 🞎**  **its not important 🞎 it is not realy important🞎** | **14.Controlling overweight and obesity and reaching a normal weight is helpful for increasing my self-confidence** |
| **quite agree🞎 agree 🞎 No idea 🞎 disagree 🞎 quite disagree 🞎** | **15.To be able to increase my self-confidence by losing weight** |
| **It is very important🞎 important 🞎 No idea 🞎**  **its not important 🞎 it is not realy important🞎** | **16.Because my parents are overweight and obese, diet and exercise have little effect on my weight loss** |
| **quite agree🞎 agree 🞎 No idea 🞎 disagree 🞎 quite disagree 🞎** | **17.The third part questions related to attitude** |
| **It is very important🞎 important 🞎 No idea 🞎**  **its not important 🞎 it is not realy important🞎** | **18.The effect of parents' overweight and obesity on my weight loss** |
| **quite agree🞎 agree 🞎 No idea 🞎 disagree 🞎 quite disagree 🞎** | **19. Because my family members use high-calorie and unhealthy foods in their daily diet and I can't lose weight.** |
| **It is very important🞎 important 🞎 No idea 🞎**  **its not important 🞎 it is not realy important🞎** | **20. The effect of the family meal plan on my weight loss** |

**Part three attitude questions**

**The fourth part: the questions related to abstract norms**

| **Please read the questions carefully and comment on each of the following sentences.** | | |
| --- | --- | --- |
| **quite agree🞎 agree 🞎 No idea 🞎 disagree 🞎 quite disagree 🞎** | 1. My parents regularly encourage me to lose weight. | |
| **quite agree🞎 agree 🞎 No idea 🞎 disagree 🞎 quite disagree 🞎** | 1. My family members encourage me to be active in my free time instead of using TV, computer and cell phone. | |
| **quite agree🞎 agree 🞎 No idea 🞎 disagree 🞎 quite disagree 🞎** | 1. My close friends expect me to lose weight. | |
| **quite agree🞎 agree 🞎 No idea 🞎 disagree 🞎 quite disagree 🞎** | 1. Most of the family members support me to lose weight and reach a suitable weight. | |
| **quite agree🞎 agree 🞎 No idea 🞎 disagree 🞎 quite disagree 🞎** | 1. My teachers think that I should not eat high-calorie foods to lose weight | |
| **quite agree🞎 agree 🞎 No idea 🞎 disagree 🞎 quite disagree 🞎** | 1. The staff of health centers approve daily regular physical activity such as walking and cycling to lose weight. | |
| **1.How important is the opinion of these people to you in reaching a normal weight?** | | |
| **Very much🞎 much 🞎 little 🞎 very little 🞎 at all 🞎** | | 2. mother |
| **Very much🞎 much 🞎 little 🞎 very little 🞎 at all 🞎** | | 3. father |
| **Very much🞎 much 🞎 little 🞎 very little 🞎 at all 🞎** | | 4. Family members, uncles, aunts, uncles |
| **Very much🞎 much 🞎 little 🞎 very little 🞎 at all 🞎** | | 5. Doctors and staff of health and treatment centers |
| **Very much🞎 much 🞎 little 🞎 very little 🞎 at all 🞎** | | 6. Clodse friend |
| **Very much🞎 much 🞎 little 🞎 very little 🞎 at all 🞎** | | 7. Teachers |

| Please read the following questions carefully and comment on each of the following sentences | |
| --- | --- |
| **quite agree🞎 agree 🞎 No idea 🞎 disagree 🞎 quite disagree 🞎** | 1.It is difficult for me to use a low calorie diet to lose weight |
| **quite agree🞎 agree 🞎 No idea 🞎 disagree 🞎 quite disagree 🞎** | 2.It is impossible for me to use a low-calorie diet to lose weight during celebrations and parties |
| **quite agree🞎 agree 🞎 No idea 🞎 disagree 🞎 quite disagree 🞎** | 3.It is difficult for me to avoid consuming snacks, chips, puffs, soft drinks, industrial juices to lose weight. |
| **quite agree🞎 agree 🞎 No idea 🞎 disagree 🞎 quite disagree 🞎** | 4.It is impossible for me to avoid snacks, chips, soft drinks, artificial juices in recreational environments with my friends |
| **quite agree🞎 agree 🞎 No idea 🞎 disagree 🞎 quite disagree 🞎** | 5.It is difficult for me to do daily physical activities instead of using TV, computer and mobile phone to lose weight |
| **quite agree🞎 agree 🞎 No idea 🞎 disagree 🞎 quite disagree 🞎** | 6.It is impossible for me to do daily physical activities instead of using TV, computer and cell phone to lose weight when I am bored. |
| **quite agree🞎 agree 🞎 No idea 🞎 disagree 🞎 quite disagree 🞎** | 8.It is difficult for me to walk or bike to lose weight instead of taking bus and taxi |
| **quite agree🞎 agree 🞎 No idea 🞎 disagree 🞎 quite disagree 🞎** | 9.Having to walk or ride a bike to lose weight instead of taking the bus and taxi when I'm tired is impossible for me |
| **quite agree🞎 agree 🞎 No idea 🞎 disagree 🞎 quite disagree 🞎** | 10.It is impossible for me to reach the ideal weight despite obstacles and problems such as not having free time for homework |
| **quite agree🞎 agree 🞎 No idea 🞎 disagree 🞎 quite disagree 🞎** | 11.Obstacles and problems cannot stop me from reaching my ideal weight |

**"Part Five: Perceived Behavioral Control Questions"**

**"** **The sixth part questions related to behavioral intention"**

| Please commented your opinion about fallowing sentences. | Behaveoral intentional |
| --- | --- |
| **quite agree🞎 agree 🞎 No idea 🞎 disagree 🞎 quite disagree 🞎** | 1.I plan to reduce the types of sandwiches, sausages, hamburgers, dumplings, snacks, and pizzas to lose weigh |
| **quite agree🞎 agree 🞎 No idea 🞎 disagree 🞎 quite disagree 🞎** | 2.I have decided to reduce the consumption of chips, puffs, soft drinks and industrial juices to lose weight |
| **quite agree🞎 agree 🞎 No idea 🞎 disagree 🞎 quite disagree 🞎** | 3.I will try to follow my weight loss diet during parties, celebrations, religious ceremonies |
| **quite agree🞎 agree 🞎 No idea 🞎 disagree 🞎 quite disagree 🞎** | 4.I plan on walking or cycling instead of using vehicles to lose weight |
| **quite agree🞎 agree 🞎 No idea 🞎 disagree 🞎 quite disagree 🞎** | 5.I am determined to lose weight by doing regular physical activity in my spare time |
| **quite agree🞎 agree 🞎 No idea 🞎 disagree 🞎 quite disagree 🞎** | 6.I plan to never overeat and snack again to lose weight |
| **quite agree🞎 agree 🞎 No idea 🞎 disagree 🞎 quite disagree 🞎** | 7.I plan to pay attention to the calories, sugar and fat content of foods when shopping at the grocery store |

|  | Behavior or performance |
| --- | --- |
| **never🞎 rarely 🞎 some times 🞎 mostly 🞎 Always 🞎** | 1.Do you use 2 to 3 units of fruit in your diet per day?Each unit of the fruit group is equivalent to one large fruit and half a glass of small fruits |
| **never🞎 rarely 🞎 some times 🞎 mostly 🞎 Always 🞎** | 2.Do you use 2 to 3 units of vegetables per day in your diet? Each unit of vegetables is equivalent to half a glass of cooked vegetables or one glass of raw vegetables |
| **never🞎 rarely 🞎 some times 🞎 mostly 🞎 Always 🞎** | 3.Do you use 2 to 3 glasses of dairy products in your diet, including milk, yogurt, buttermilk, and cheese? |
| **never🞎 rarely 🞎 some times 🞎 mostly 🞎 Always 🞎** | 4.Do you use fish in your diet once or twice a week? |
| **never🞎 rarely 🞎 some times 🞎 mostly 🞎 Always 🞎** | 5.Do you use healthy snacks and oily nuts like walnuts, pistachios, and almonds in small amounts instead of low-value snacks like chips, puffs, and carbonated drinks? |
| **never🞎 rarely 🞎 some times 🞎 mostly 🞎 Always 🞎** | 6.Do we use low-calorie foods instead of pizza, sandwiches, sausages, hamburgers, dumplings, snacks, etc? |
| **never🞎 rarely 🞎 some times 🞎 mostly 🞎 Always 🞎** | 7.Do you observe your diet in parties, celebrations, religious ceremonies, etc? |
| **never🞎 rarely 🞎 some times 🞎 mostly 🞎 Always 🞎** | 8.Do you walk or bike instead of using vehicles? |
| **never🞎 rarely 🞎 some times 🞎 mostly 🞎 Always 🞎** | 9.Do you do 30 to 60 minutes of regular physical activity in your spare time? |
| **never🞎 rarely 🞎 some times 🞎 mostly 🞎 Always 🞎** | 10.When buying food, do you pay attention to its calories, sugar and fat content? |

**"** **This questionnaire is not for testing, so the questions are not graded and there are no right or wrong answers. Please answer all questions honestly and accurately."**

**International Physical Activity Questionnaire**

Please answer the following questions about the types and amount of physical activity you did during the past seven days.

1. **During the last seven days, how many days have you had intense physical activity such as lifting heavy objects and aerobic values ​​such as cycling, running, football, volleyball, etc**.
   1. day of the week
   2. I have not been very active
2. **If you did vigorous activity, how long did you do vigorous physical activity during those days?**
3. hours per day
4. minutes per day

1. **During the past seven days, how many days have you done moderate-intensity physical activity other than walking, such as light lifting, bicycling, jogging, or doubles tennis**

A. day of the week

B. I have not had an average activity

1. **If you do moderate activity, how long have you been doing vigorous physical activity during those days?**
   1. hours per day
   2. minutes per day
2. **How many days have you walked for at least 10 minutes during the past seven days?**
3. day of week
4. I have not walked at all
5. **If you have a walk, how long was it on those days?**
6. hours per day
7. minutes per day
8. **During the past seven days, how much time per day did you spend sitting, such as sitting in front of the TV, computer, or reading?**
9. hours per day
10. minutes per day
